# Flaxseed oil and alpha-lipoic acid combination ameliorates hepatic oxidative stress and lipid accumulation in comparison to lard

**DOI:** 10.1186/1476-511X-12-58

**Published:** 2013-05-01

**Authors:** Jiqu Xu, Hui Gao, Lin Song, Wei Yang, Chang Chen, Qianchun Deng, Qingde Huang, Jin’e Yang, Fenghong Huang

**Affiliations:** 1 Department of Product Processing and Nutriology, Oil Crops Research Institute, CAAS, 2 Xudong Second Road, Wuhan 430062, P.R. China; 2 Hubei Key Laboratory of Lipid Chemistry and Nutrition, 2 Xudong Second Road, Wuhan 430062, P.R. China; 3 Department of Nutrition and Food Hygiene, School of Public Health, Tongji Medical College, Huazhong University of Science and Technology, 13 Hangkong Road, Wuhan 430030, P.R. China; 4 Department of neurology, Hubei Xinhua Hosipital, 11 lingjiaohu Road, Wuhan 430015, P.R. China; 5 Department of Gastroenterology, The First People's Hospital of Yichang, The People's Hospital of China Three Gorges University, 2 Jiefang Road, Yichang 443000, P.R. China

**Keywords:** Flaxseed oil, α-lipoic acid, High fat diet, Lipid accumulation, Oxidant stress

## Abstract

**Background:**

Intake of high-fat diet is associated with increased non-alcoholic fatty liver disease (NAFLD). Hepatic lipid accumulation and oxidative stress are key pathophysiological mechanisms in NAFLD. Both flaxseed oil (FO) and α-lipoic acid (LA) exert potential benefit to NAFLD. The aim of this study was to determine the effect of the combination of FO and LA on hepatic lipid accumulation and oxidative stress in rats induced by high-fat diet.

**Methods:**

LA was dissolved in flaxseed oil to a final concentration of 8 g/kg (FO + LA). The rodent diet contained 20% fat. One-fifth of the fat was soybean oil and the others were lard (control group), or 75% lard and 25% FO + LA (L-FO + LA group), or 50% lard and 50% FO + LA (M-FO + LA group), or FO + LA (H-FO + LA group). Male Sprague–Dawley rats were fed for 10 weeks and then killed for liver collection.

**Results:**

Intake of high-fat lard caused a significant hepatic steatosis. Replacement with FO + LA was effective in reducing steatosis as well as total triglyceride and total cholesterol contents in liver. The combination of FO and LA also significantly elevated hepatic antioxidant defense capacities, as evaluated by the remarkable increase in the activities of SOD, CAT and GPx as well as the level of GSH, and the significant decline in lipid peroxidation.

**Conclusion:**

The combination of FO and LA may contribute to prevent fatty livers such as NAFLD by ameliorating hepatic lipid accumulation and oxidative stress.

## Introduction

Nonalcoholic fatty liver disease (NAFLD) is a clinicopathological term that encompasses a broad spectrum ranging from benign hepatic steatosis to cirrhosis. It is the most common liver disease and now recognized as a major public health problem in contemporary society around the world [1,2]. High-fat diet is the most common cause of NAFLD [3] because chronic consumption of this type of diet leads to obesity, abnormalities of lipid metabolism and insulin resistance, all of which have been reported to be linked to NAFLD [4]. Regardless of etiology of NAFLD, lipid accumulation and oxidative stress are two requisite for this disease progression [5].

Flaxseed oil (FO) is a particularly rich source of α-linolenic acid (LNA) with concentrations ranging from approximately 40% to 60% [6]. As a nutritionally essential polyunsaturated fatty acid (PUFA), LNA can act as the precursor of longer chain n-3 PUFA (EPA and DHA) or compete with linoleic acid to reduce arachidonic acid content or direct interaction with ion channels and nuclear receptors, and thus may exert numerous beneficial effects in the human body, such as antiarrhythmic, antiinflammatory and neuroprotective functions as well as accelerating brain growth in preterm and neonates [7]. Recently, LNA has been reported to lower serum lipids, liver size and hepatic lipids contents and thus attenuate NAFLD [8]. However, since LNA is highly susceptible to oxidation, FO addition may result in a significantly higher lipid peroxidation in liver and other tissues [9], which may have an adverse effect on hepatoprotection.

α-lipoic acid (LA), also referred to as thioctic acid, is a disulfide compound that is found naturally in mitochondria as the coenzyme for pyruvate dehydrogenase and α-ketoglutarate thus serves a critical role in mitochondrial energy metabolism. Although orally supplied LA may not be used as a metabolic cofactor, there is a unique set of biochemical activities with potential pharmacotherapeutic value against a host of pathophysiologic insults [10]. For example, LA has gained considerable attention as an excellent antioxidant to reduce oxidative stress [11-13]. Further, LA is fat- and water-soluble, which makes it effective against a broader range of free radicals. It has also been demonstrated recently that LA has ability to prevent hepatic steatosis in rat fed a long-term high-fat diet [14]. These beneficial effects make LA possess the potential abilities to improve NAFLD [15].

The combination of FO and LA has been demonstrated to reduce atherosclerosis risk in our previous study [16]. The purpose of the present study was to determine whether the combination of FO and LA is able to decrease hepatic lipid accumulation and oxidative stress in rats fed a high-fat diet.

## Materials and methods

### Chemical sources

Commercial deodorized lard and soybean oil (food grade) was purchased from a local supermarket. The flaxseed oil (food grade) was obtained from Caoyuankangshen Food Co., Ltd (Inner Mongolia, China). The fatty acids compositions of these experimental oils are shown in Table 1. LA ((±)-α-Lipoic acid) was purchased from Sigma-Aldrich (St. Louis, MO, USA) and was dissolved in flaxseed oil to a final concentration of 8 g/kg (FO + LA) when used.

**Table 1:** **Fatty acid composition of the experimental oils (%)**^**1**^

**Fatty acid**	**Lard**	**Soybean oil**	**Flaxseed oil**
C14 : 0	1.36	0.078	ND^2^
C16 : 0	29.32	10.77	6.13
C16 : 1	2.21	0.05	ND
C18 : 0	13.31	4.00	3.67
C18 : 1	42.81	24.77	22.49
C18 : 2	9.67	52.62	14.86
C18 : 3	0.27	5.94	52.84
C20 : 0	0.58	0.36	ND
C20 : 1	0.47	0.29	ND
C22 : 0	ND	0.38	ND

^1^ Values given in this table are the means of three determinations.

^2^ ND = Not detected.

### Animals and diets

The experiment was conducted with 40 male Sprague–Dawley rats (initially weighing 150–170 g). The animals were housed individually and maintained at a controlled ambient temperature (24 ± 1°C) under diurnal conditions (light–dark: 08:00–20:00) with access to laboratory chow and tap water ad libitum. After 1 week of acclimatization, rats were randomly divided into control (CON) group and three experimental groups (n =10 per group). All animals were fed purified experimental diets which contained 35% maize starch, 20% casein, 15% sucrose, 5% cellulose, 3.5% mineral mixture (AIN-93M), 1% vitamin mixture (AIN-93M), 0.3% DL-methionine, 0.2% choline bitartrate and 20% fat. One-fifth of the fat in the diet of each group was provided by soybean oil to avoid essential fatty acid deficiency, and the remaining were either lard (CON group), or 75% lard and 25% FO + LA (L-FO + LA group), or 50% lard and 50% FO + LA (M-FO + LA group), or FO + LA (H-FO + LA group). Every week, all ingredients for the purified diets were mixed, formed into a dough with purified water, rolled into pellets, sealed in air-tight plastic bags under nitrogen gas and stored at −80°C until use. The food in the animal cages was shaded from light and changed every day. The animals were cared for in accordance with *the Guiding Principles in the Care and Use of Animals*. The experiment was approved by the Oil Crops Research Institute Council on Animal Care Committee, Chinese Academy of Agricultural Sciences.

### Tissue preparation

After 10 weeks of treatment, rats were fasted for 16 hours and then killed under anaesthesia. Liver was rapidly dissected, weighed, and a small piece of right liver lobe was fixed in 4% paraformaldehyde for hematoxylin-eosine staining. The remaining liver tissue was stored at −80°C until analysis.

### Assay of liver antioxidant capacity and lipid peroxidation

Superoxide dismutases (SOD) activity was measured according to the method of Kono [17]. Catalase (CAT) activity was estimated basing on the method of Goth [18]. Glutathione peroxidase (GPx) activity was measured by the method of Sazuka [19]. The glutathione (GSH) content was determined by the method of Moron [20]. Thiobarbituric acid reactive substances (TBARS) level was estimated by the method of Buege and Aust [21]. The detection procedure of these enzymes activities has been described in detail in our previous report [22].

### Assay of protein concentration

The protein concentration was determined according to the method of Lowry [23], using bovine serum albumin (BSA) as standard.

### Assay of liver lipid content

Lipids were extracted from 1 g liver with a mixture of chloroform/methanol (2:1, v/v) by the method of Folch [24]. Total triglyceride (TG) and total cholesterol (TC) contents were measured with commercial kits (Zhongsheng Beikong Biotech Company, China).

### Statistical analyses

Results were expressed as mean ± SEM (standard error of the mean). Statistical analysis were based on one-way ANOVA, followed by the Fisher PLSD post hoc test if the overall differences were significant. All statistical analyses were performed using SPSS 13.0 statistical software (SPSS Inc., Chicago, IL) and the limit of statistical significance was set at p < 0.05.

## Results

### Food intake and body weight gain

No significant differences were observed in food intake or in body weight gain among all groups. The body weight was 162.1 ± 3.5, 163.7 ± 3.1, 160.5 ± 2.9, 162.9 ± 2.5 g at the beginning and 454.0 ± 5.7, 446.8 ± 6.8, 437.1 ± 5.3, 443.2 ± 4.1 g at the end of the study for groups 1–4, respectively.

### Effects of FO and LA combination on liver morphology

**Figure 1:**
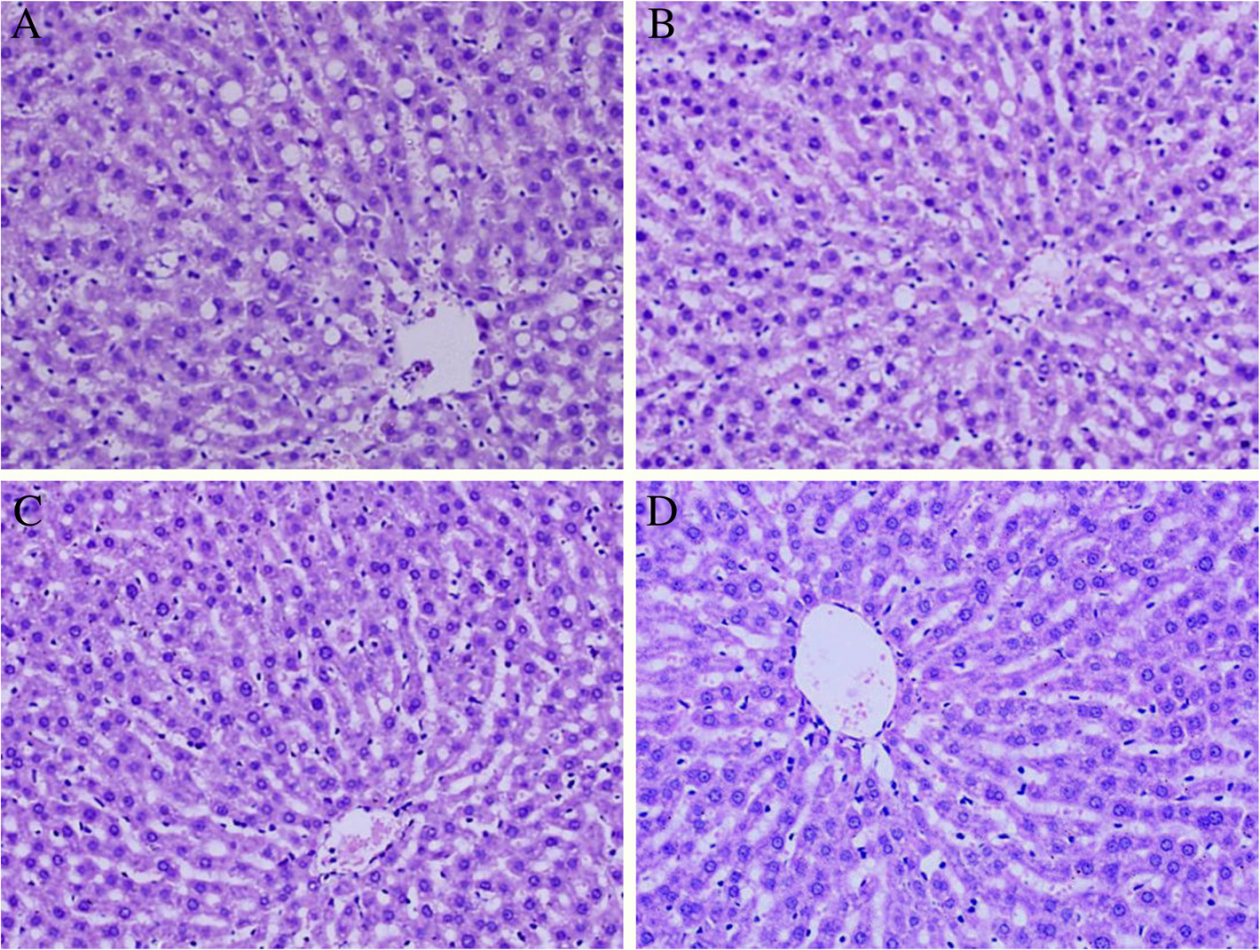
**Liver histology after hematoxylin-eosine staining of liver sections from a representative rat from each group. A**: control group; **B**: low contents of FO and LA combination group; **C**: middle contents of FO and LA combination group; **D**: high contents of FO and LA combination group.

As shown in Figure 1, HE–stained liver sections pointed that the chronic high-fat lard diet induced significantly increased macrovesicular steatosis. The circular lipid droplets in both number and size were markedly reduced in L- and M-FO + LA livers and even not present in H-FO + LA livers.

### Effects of FO and LA combination on liver lipids content

**Figure 2:**
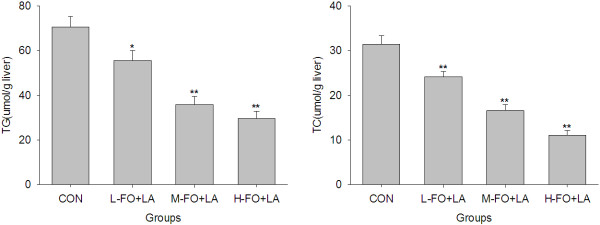
**Effects of FO and LA combination on hepatic TG and TC contents of rats fed a high-fat diet.** CON: control group; L-. M- and H- FO + LA: low, middle and high contents of FO and LA combination groups. Bars represent the mean ± SEM from 10 animals in each group. * *p* < 0.05 and ** *p* < 0.01 compared to the control group.

There are not significant differences in liver weights among all groups. However, consistent with histologic assessment, and as can be seen from Figure 2, consumption of all three doses of the combination of FO and LA significantly reduced hepatic lipids accumulation in contrast with the high-fat lard diet, as evidenced by the markedly lower TG and TC contents of livers in all FO + LA groups than in control group.

### Effects of FO and LA combination on liver antioxidative capacity and lipid peroxidation

**Figure 3:**
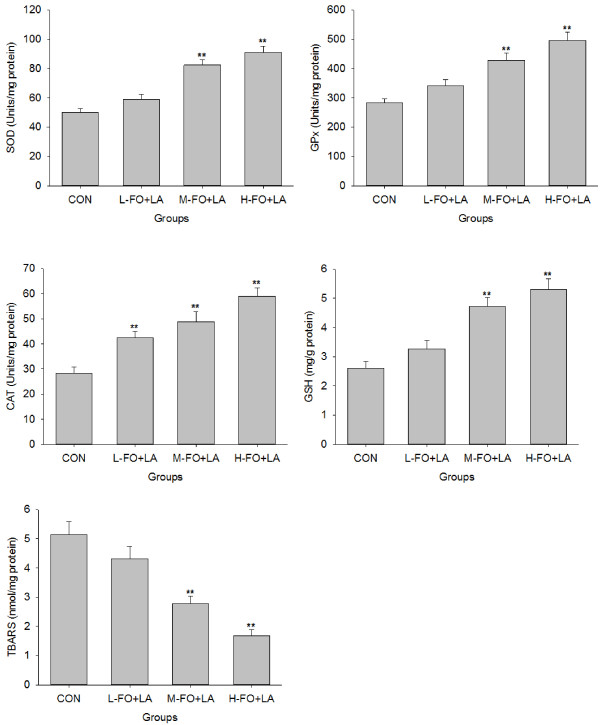
**Effects of FO and LA combination on the activities of antioxidant enzymes (SOD, GPx and CAT), the levels of GSH and the contents of TBARS in liver of rats fed a high-fat diet.** CON: control group; L-. M- and H- FO + LA: low, middle and high contents of FO and LA combination groups. Bars represent the mean ± SEM from 10 animals in each group. ** *p* < 0.01 compared to the control group.

As shown in Figure 3, the activities of hepatic antioxidant enzymes SOD and GPx were found to be statistically elevated in M- and H- FO + LA animals, as compared to high-fat lard animals. In addition, the CAT activities in livers of all FO + LA groups were significantly higher than control group. Compared with high-fat lard animals, the levels of hepatic GSH were remarkably increased in M- and H- FO + LA administered rats. When plasma TBARS were evaluated as the marker of lipid peroxidation, noticeable declines in the TBARS contents were seen in the livers of M- and H- FO + LA groups.

## Discussion

NAFLD covers a wide disease spectrum of liver pathology, ranging from simple hepatic steatosis to nonalcoholic steatohepatitis (NASH) and cirrhosis. The simple steatosis is generally nonprogressive and reversible, while NASH is increasingly recognized as a precursor to more severe liver disease and may develop progressive hepatic fibrosis and cirrhosis [2]. Although the pathogenesis of NAFLD has not yet been fully elucidated, a most widely accepted mechanism is the “two-hit” theory [5]. The “first hit” is excessive hepatic fat accumulation, a process that is closely linked with insulin resistance, and the “second hit” is hepatocellular injury that results from oxidative stress. High-fat diet can induce significant insulin resistance [25] as well as oxidative stress in liver [26], and thus induces the development of NAFLD [27].

Histologic evaluation is considered the “gold standard” in evaluation of the presence and severity of NAFLD [28]. In the present study, the livers of the animals fed a high-fat lard diet show noticeable hepatic steatosis. The combination of FO + LA significantly improved the severe hepatic steatosis, which meant that this combination can protect against NAFLD. In parallel with the changes in liver morphology, all of the L-, M- and H-FO + LA reduced markedly both hepatic TG and TC contents. The main components FO and LA in the combination were both responsible for these beneficial changes. LNA has been well established to be a natural ligand of peroxisome proliferator-activated receptor-α (PPARα) [29] and shown to bind and activate this key transcriptional regulator of lipid metabolism, thereby increases the gene expression and activities of enzymes involved in fatty acid oxidation in the liver [30-33]. LNA itself is a better substrate for the mitochondrial and peroxisomal β-oxidation pathways thus stimulating oxidation of fatty acid in liver [33]. PPARγ, another member of the PPAR family, is also a major transcriptional regulator of lipid metabolism. FO has been considered to induce a notable increase in the hepatic mRNA expression of PPARγ which is negatively correlated with hepatic lipid levels [34]. In addition, the depletion of hepatic n-3 long-chain polyunsaturated fatty acids (LCPUFA) is proposed to be a major factor contributed to liver steatosis found in NAFLD [35]. As precursor fatty acid of n-3 LCPUFA, LNA is able to increase significantly EPA and DHA contents in hepatic membrane and thus declines hepatic lipid levels [36]. Besides, in response to LNA rich FO, the higher activity of CYP7A1 results in a higher cholesterol secretion into bile and then lowers the intrahepatic pool of cholesterol [37]. Consistent with a microarray analysis [38], LA has been reported to increase mRNA level of PPARα [14], and as a consequence, up-regulate the gene expression of several enzymes and proteins involved in mitochondrial β-oxidation especially long-chain acyl coenzyme A dehydrogenase (LCAD) [14], a key enzymes necessary for regulation of mitochondrial β-oxidation of fatty acids and for the development of hepatic steatosis [14]. Also, LA may reduce the acetylation level of LCAD and then induce its enzymatic activity directly through stimulation of SIRT3 [39,40]. On the other hand, LA has been reported to decrease the mRNA levels [14,41] and activities [41] of a series of enzymes involved in hepatic fatty acid synthesis such as fatty acid synthase and ATP-citrate lyase. Moreover, LA intake also inhibits liver cholesterol synthesis by down-regulating the expression of genes for enzymes involved in cholesterol synthesis [38].

High-fat diet reduces the levels of hepatic antioxidants, which results in the remarkable increase in oxidative stress and lipid peroxidation [42,43]. In the present study, administration of FO + LA combination significantly elevated the activities of antioxidant enzymes SOD, CAT and GPx as well as level of GSH. The increased antioxidant capability in liver combining with the remarkable decline of hepatic TBARS levels meant that FO + LA consumption significantly attenuated oxidative stress in liver. Although FO might decrease the depletion of hepatic GSH contents by lowering hepatic cholesterol and triacylglycerol levels [8], the drastic hepatic oxidative stress improvement effect of FO + LA consumption in this study should mainly attribute to the antioxidant properties of LA. LA is an eight-carbon structure that contains a disulfide bond as part of a dithiolane ring, and this characteristic structure is responsible for the potent antioxidant activity of this molecule and its reduced form, dihydrolipoic acid (DHLA). In fact, both LA and DHLA are capable of scavenging a variety of free radicals and also chelating redox active transition metals [10]. Far longer than its sole chemical reduction and redox properties, LA may maintain hepatic low molecular weight antioxidants vitamin C and GSH levels by inducing the uptake or enhancing the synthesis [10]. Also, this molecule may act as a transient but potent mediator of stress response signaling and thus affects other important antioxidants [44]. For example, LA can increase the activities of SOD and GPx in liver of rats fed high fat diet through the stimulation of SIRT1 and SIRT3 [40]. In addition, LA appears to be an inducer of Nrf2-mediated antioxidant gene expression [44,45], which significantly increases expression of the antioxidant enzyme heme oxygenase-1 [15] and cellular capacity to synthesize GSH in liver [44]. In response to aforementioned antioxidative mechanisms, LA plays a critical role in determining antioxidative capacity of FO and LA combination and may be mainly responsible for the substantially improved hepatic oxidative stress in this experiment.

## Conclusions

In summary, substitution of lard with FO + LA reduced triacylglycerol and cholesterol levels and elevated the capacity of antioxidant defense in liver in a dose-dependent manner, which meant that this combination has the ability to reduce excessive hepatic fat accumulation and oxidative stress. These results indicated that the combination of FO + LA might contribute to ameliorate nonalcoholic fatty liver induced by high-fat lard diet.

## Competing interests

The authors declare that they have no competing interests.

## Authors’ contributions

Author JX designed and wrote a first draft of the paper. HG, SR, WY, QD and JY carried out all the experiments. CC participated in the design of the study. QH performed the data analysis and created the figures. FH contributed to the design of the study, reviewed the manuscript and contributed to the final version. All authors contributed to and have approved the final manuscript.
